# E-Bike Injuries: Experience from an Urban Emergency Department—A Retrospective Study from Switzerland

**DOI:** 10.1155/2014/850236

**Published:** 2014-03-20

**Authors:** Sylvana Papoutsi, Luca Martinolli, Christian Tasso Braun, Aristomenis K. Exadaktylos

**Affiliations:** Department of Emergency Medicine, University Hospital and University of Bern, Freiburgstraße 16c, 3010 Bern, Switzerland

## Abstract

*Background*. Between 2005 and 2012, annual sales of E-bikes in Switzerland increased from 1,792 to 52,941. This continuous and rapid transition from human-powered bicycles to an electric bicycle technology may indicate the increasing demand for low-cost transportation technology in combination with a healthy lifestyle. * Material and Methods*. In the present study, from April 2012 to September 2013, we retrospectively analysed E-bike accidents treated in the Emergency Department of our hospital by focusing on the following parameters: age, gender, time, period, and cause of the accident, as well as injury and outcome. * Results*. Patients were predominantly male. The mean age of injured E-cyclists was 47.5 years. The main causes of injury were self-accident. Most injuries were to the head/neck. The mean ISS was 8.48. The outcome showed that 9 patients were treated as outpatients, 9 were inpatients, and 5 patients were kept in the Intensive Care Unit (ICU). Only six patients underwent surgery (S). * Discussion*. This is the first attempt to evaluate E-bike injuries in Switzerland in an acute hospital setting. Since there is increasing popular preference for E-bikes as means of transportation and injuries to the head or neck are prevalent among E-cyclists, the hazard should not to be underestimated.

## 1. Introduction

Electric bikes or E-bikes are a category of modern vehicles that includes two-wheel bikes propelled by human pedalling, supplemented by electrical power from a storage battery (bicycle-style). The main technical components include a hub motor and lead-acid (VRLA) battery regulated by a controlled valve. Bicycle-style E-bikes typically have 36 V batteries and 180–250 W motors. E-bikes are regulated not to exceed 20 km/h on electric power alone; however, many E-bikes can travel at speeds in excess of that limit and some are advertised as being able to reach 40 km/h and more [1].

Approximately 29 million E-bikes were produced in 2010, representing a 24.7% increase over 2009 [2]. By 2011, 120 million E-bikes had been registered in China [3], which might account for 90% of the global market [2]. Furthermore, a worldwide increase in demand is expected; it is estimated that 466 million E-bikes will have hit the road by 2016 [4].

These vehicles have also become a popular transportation mode for the Swiss [5], as they provide a long-term, less expensive, and convenient form of private mobility and are thus an attractive alternative to public transport, private cars, motorbikes, or normal cycling. Due to their low energy consumption and almost zero emissions [6], these vehicles are promoted by local governments [7]. They are safer than motorcycles, more comfortable and cheaper than the bus, and cheaper than the car. Longer distances can be travelled than with normal bikes [8]. Thus, from the user's perspective, E-bikes offer many advantages over regular bikes and walking.

As was mentioned before, E-bikes are gaining an increasing share of two-wheel transportation. Swiss statistics have shown that in 2005 there were a total of 1,792 E-bikes. The latest statistics from the Swiss Institute for Bicycles (SFZ) indicate that E-bike sales in Switzerland have “sky rocketed,” with 52,941 annual E-bike sales and an annual rate of increase of 15.2% (Table 1) [5].

Notwithstanding the huge increase in the number of E-cyclists, the literature on E-bikes injuries is relatively limited. We have now performed the first retrospective study of E-bike injuries at a European level I trauma centre.

## 2. Material and Methods

Our University Department of Emergency Medicine, the only Level I centre in the canton of Bern, serves about 1,8 million people and treats more than 35,000 cases per year, caring for patients older than 16 years. Our retrospective 18-month observational study, from April 2012 to September 2013, comprised adult patients (≥16 years old) admitted to our Emergency Department (ED) in relation to an accident that had occurred with an E-bike. All patients presenting to the ED with an E-bike accident during the study period were included in our study. Patients were identified using the appropriate search string in the patient demographic field of our computerised patient database. Since this medical database allows instantaneous recall of past diagnostic reports, consultations, X-rays, and other relevant medical documents, the authors retrospectively analysed the E-bike injured patients, including gender, age (<40 or ≥40 years old), time (morning 05:01 am–13:30 pm, afternoon 13:31 pm–17:30 pm, evening 17:31 pm–21:00 pm, and night 21:01 pm–05:00 am), season (spring, summer, autumn, and winter), and cause of accident: being caught in a tram rail (occurred after being caught in a tram rail), vehicle collision (occurred after collision with a vehicle), and self-accident (occurred due to alcohol intoxication, due to high speed, or without a known reason), as well as severity of injury, medical diagnosis, and, finally, the outcome (treated as home/outpatient, kept in hospital, or as inpatient, kept in the Intensive Care Unit and mortality). Each diagnosis was classified according to the Abbreviated Injury Scale (AIS) handbook 2008 [9] and the Injury Severity Score (ISS) was calculated for each patient [10].

## 3. Results 

During the 18-month study period, from April 2012 until September 2013, a total of twenty-three (*n* = 23) E-bike accidents were reported to our ED. Seventy percent (*n* = 16) of the injured were male and 30% (*n* = 7) female. A total of 18 (78.3%) of the injured E-cyclists were above the age of 40. The median age was 47.5 years. The detailed characteristics of each patient are shown in Table 2.

Additionally, 43.5% (*n* = 10) of accidents took place during the morning, 26.1% (*n* = 6) during the afternoon, 17.4% (*n* = 4) during the evening, and only 13.0% (*n* = 3) during the night. The most common season was summer, with 39.1% (*n* = 9) of accidents, followed by spring and autumn, each with 26.1% (*n* = 6), as well as winter with 8.7% (*n* = 2). Approximately 60.9% (*n* = 14) of injured E-cyclists were involved in a self-accident of known/unknown cause: these were related to a high speed (*n* = 4; 17.4%), to alcohol intoxication (*n* = 2; 8.7%), or no known reason (*n* = 8; 34.8%). Furthermore, 5 (21.7%) E-cyclists were injured after being caught in a tram rail and 4 (17.4%) due to a collision with a vehicle.

**Figure 1:**
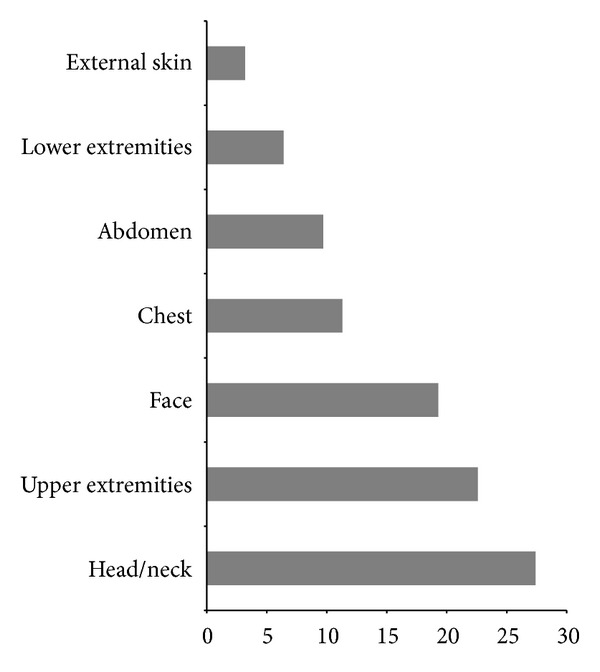
Percentage of injured regions among E-cyclists (%).

**Figure 2:**
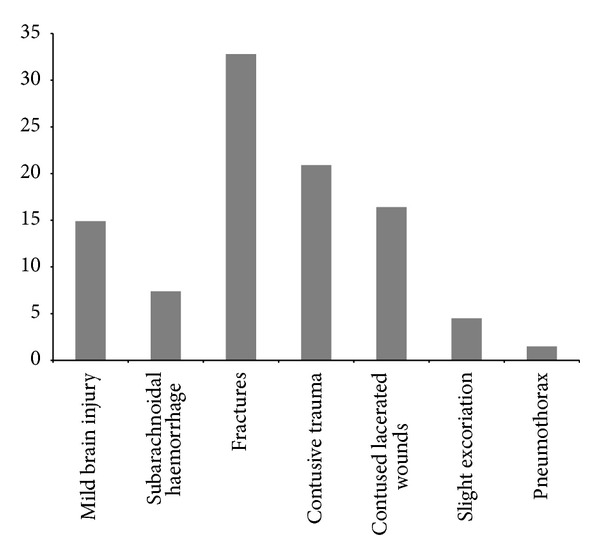
Type of injuries in E-cyclists in percentage (%).

The head/neck was the most common body region injured (27.4%). Injuries to the upper extremities (22.6%) and face (19.3%) were almost as common. Injuries to the chest (11.3%), abdomen (9.7%), lower extremities (6.4%), and external skin (3.2%) were less frequent (Figure 1). The types and locations of the injuries are summarised in Table 3. In particular, the most common injuries to the head/neck were mild brain injury (14.9%) and subarachnoid haemorrhage (7.4%); to the face, contused lacerated wounds (10.4%) and fractures (4.5%); to the upper extremities, clavicle fractures (11.9%) and contusive trauma (8.9%); to the chest, rib fractures (6.0%); to the low extremities, contusive trauma (4.5%); to the abdomen, contusive trauma (3.0%); to the external skin, (4.5%) slight excoriation (without contusive trauma). The most frequent types of injuries were fractures (32.6%)—especially fractures to the clavicle (38.1%)—contusive trauma (20.9%), and mild brain injuries (14.9%) (Figure 2).

The ISS scores were between 2 and 29, with an average value of 8.48. Of the 14 (60.8%) hospitalised patients, only 6 (42.8%) underwent surgery.

Only 9 patients (39.1%) were sent home or for outpatient treatment, but 14 patients (60.81%) were treated in hospital. In the latter group, 9 patients (39.1%) were treated in general wards (3 patients in the Oral/Maxillofacial Unit, 3 in the Neurosurgery Unit, 2 in the Orthopaedics/Traumatology Unit, and 1 in the Dermatology Unit) and the remaining 5 patients (21.7%) were kept in ICU. No E-cyclist died.

## 4. Discussion

The current retrospective 18-month descriptive study aims to make a preliminary evaluation of E-bike injuries, in the Emergency Department (ED) of an acute Level I Trauma Centre in Switzerland. To the best of our knowledge, this is one of the first studies that systematically analyses the accidents related to this means of transport at a European hospital.

Our results showed that E-bike users are predominantly male; this finding is in full concordance with the current international literature [11–15]. Among accident victims, 78.3% are ≥40 years old, with a median age of 47.5. Our finding with regard to the median age is in good agreement with a recent report (SINUS-Report 2013) from The Swiss Council for Accident Prevention [16]. Du et al., in a Chinese study of 323 E-bike injuries, reported an average age of 43.8 years [15]. However, some of their patients were children. More and more elderly adults in Switzerland choose E-bikes for transportation, perhaps because this takes less time and is less exhausting than riding a conventional bike. In Australia, another study reported that using an E-bike rather than a conventional bike increased the likelihood of preferring an active method of transport, and was significantly quicker, with less perceived effort [17].

The present study also showed that most E-bike accidents occurred during the morning, followed by evening and afternoon, as well as in the summer months. Du et al. also analysed the time of the day and concluded that most accidents occurred during the night (18:00 pm–23:59 pm). Our findings are consistent with peak travel to and from work; they may indicate the use of E-bikes as a means of transportation to and from work.

Furthermore, we noticed that most E-bike injuries were caused by self-accident and a significant percentage of accidents were attributed to being caught in a tram rail. It is worth mentioning that we analysed the tram rails as a cause of accident as these were very frequent among our patients. In the study of Du et al., it was reported that 48.6% of injuries involved collisions with motor vehicles. In contrast, we found that only 17.4% of accidents were caused by collision with vehicles. This may be related to the traffic density in the two countries.

Another Chinese study found that E-bike related casualties had increased in recent years, notwithstanding the decreases in the total number of road traffic collisions and deaths [18]. In Switzerland, the proportion of E-bikes in cycling traffic is rising steadily. In 2010, only 1.5% of cyclists rode E-bikes, with increases to 2.3% in 2011 and 3.8% in 2012 (Swiss Council for Accident Prevention). Unfortunately, no detailed data for E-bike injuries are available for Switzerland for these years.

The injuries to the head/neck region included a high proportion of mild brain injury. In the whole body, the most common injuries were fractures and contusions, followed by contused lacerated wounds and superficial excoriations (Figure 2). Du et al. made similar findings with respect to the head/neck region; however, the most common injuries they found were fractures of the limbs, whereas in our study, they were clavicle fractures. Moreover, the second most commonly injured body region in our study was the upper extremities (Figure 1). This is in contrast to Du et al., who recorded 46.4% of injuries in the head/neck and 20.7% in the trunk. These may relate to the failure of more E-cyclists to wear helmets in China. Indeed, the rate of helmet wearing among Swiss E-cyclists is currently 75.0% (Swiss Council for the Accident Prevention) in Switzerland [16] and only 9.0% in China [15]. Considering that the impacts and hazards are likely to be similar in the two countries, the evidence and the effectiveness of helmets in reducing bicyclist [19] and motorcyclist [20] head injuries clearly support helmet use for E-cyclists.

It is remarkable that we found no hospital mortality and our ISS score was relatively low. Unfortunately, no ISS scores on E-bike injuries have been published in comparable studies. Only eight E-bike deaths were reported in 2012 (Swiss Federal Roads Authority), which was six more than in the previous year [16]. However, Du et al. failed to find any hospital fatality in the course of their 6-month study in China. In another study carried out in Hangzhou, with its population of circa 7,800,000, Feng et al. recorded 397 E-bike related deaths between 2004 and 2008 [18].

In their retrospective study on motorcycle and bicycle accidents, Wagner et al. concluded that these accidents cause major injuries in older patients with more severe ISS. They concluded that fewer older bicyclists used helmets and that they suffered more sustained or severe head injuries, with greater functional decline [21]. An Australian study to evaluate the factors influencing the outcome of 208 motorcycle crash victims showed that losing control due to intoxication, collision with another vehicle, or travelling above the speed limit was associated with the worst patient outcome; travelling in excess of 50 kph increased the risk of intracranial injury [22]. Our patient group was too small to allow similar conclusions. 

*Limitations*. Our findings have to be considered with some caution, as the study was conducted retrospectively. As the information in our medical history database is presented in a narrative comment, there is no guarantee that the number of E-cyclists was fully reported. Furthermore, our study was limited to adults (≥16 years old), as children are treated at a separate Emergency Department in our hospital. Since the weather conditions during each accident were not reported, as a first approximation, we analysed our data by season. It has been demonstrated that helmets protect riders of two-wheel vehicles and so correlate negatively with admission to an ICU [23, 24]. Unfortunately, our register data did not document helmet use.

## 5. Conclusions

E-bikes are a new mode of transport; prices are coming down and purchases will probably continue to rise. Injuries to the head and neck are common. Further studies are needed to compare accidents suffered by E-cyclists with those suffered by bicyclists and motor cyclists.

## Figures and Tables

**Table 1:** Overview of Switzerland's bicycle market [5].

Year	Total bike sales	E-bike sales	Proportion %
2005	280,840	1,792	+0.6%
2006	299,286	3,181	+1.1%
2007	314,161	5,825	+1.9%
2008	314,784	11,631	+3.7%
2009	349,903	23,886	+6.8%
2010	351,003	39,247	+11.2%
2011	351,808	49,615	+14.1%
2012	348,643	52,941	+15.2%

**Table 2:** E-cyclists' characteristics.

Patients (*n*)	Sex	Age	Time	Period	Cause of accident	Region of injury	ISS	Outcome
1	M	38	Evening	Summer	Being caught in a tram rail	Head/neck, face, upper extremities, external	6	Home
2	M	63	Night	Spring	Self-accident (alcohol intoxication)	Head/neck, face, upper extremities, abdomen	12	Hospital
3	F	41	Evening	Spring	Self-accident (unknown reason)	Upper extremities, chest, abdomen, external	3	Home
4	M	51	Afternoon	Summer	Being caught in a tram rail	Head/neck, face, upper extremities	3	Home
5	M	54	Afternoon	Spring	Self-accident (unknown reason)	Head/neck, face, chest	17	Hospital
6	M	45	Afternoon	Spring	Collision with vehicle	Face, upper extremities, chest, lower extremities	6	Hospital
7	F	27	Afternoon	Summer	Self-accident (high speed)	Head/neck, face, upper extremities, abdomen	6	Hospital/S*
8	F	41	Morning	Autumn	Self-accident (high speed)	Lower extremities	4	Home
9	F	57	Afternoon	Autumn	Self-accident (unknown reason)	Head/neck, face, upper extremities	9	Hospital
10	F	43	Morning	Autumn	Self-accident (unknown reason)	Head/neck, chest	10	ICU**
11	M	42	Morning	Summer	Collision with vehicle	Upper extremities, chest, lower extremities	3	Home
12	M	76	Afternoon	Autumn	Collision with vehicle	Head/neck, chest, upper extremities, abdomen	29	ICU/S
13	M	59	Night	Autumn	Self-accident (unknown reason)	Head/neck, face	17	ICU/S
14	M	48	Morning	Autumn	Being caught in a tram rail	Face, chest	5	Home
15	F	64	Morning	Winter	Self-accident (unknown reason)	Head/neck, face	4	Hospital
16	F	35	Morning	Winter	Self-accident (unknown reason)	Head/neck, face, upper extremities	6	Hospital/S
17	M	62	Morning	Spring	Collision with vehicle	Upper extremities, lower extremities	13	Hospital/S
18	M	40	Evening	Spring	Self-accident (high speed)	Head/neck, face, upper extremities	5	Home
19	M	61	Morning	Summer	Self-accident (high speed)	Head/neck	9	ICU
20	M	21	Morning	Summer	Self-accident (unknown reason)	Head/neck	1	Home
21	M	41	Morning	Summer	Being caught in a tram rail	Head/neck, upper extremities, abdomen	6	Hospital/S
22	M	46	Evening	Summer	Being caught in a tram rail	Head/neck, upper extremities	8	Home
23	M	37	Night	Summer	Self-accident (alcohol intoxication)	Head/neck, abdomen	13	ICU

*Surgery; **Intensive Care Unit.

**Table 3:** Number and type of injuries for each body region.

Head/Neck	MBI* *n* = 10 (14.9%)	SAH** *n* = 5 (7.4%)	Fractures *n* = 4 (6.0%)	CLW***n = 3 (4.5%)

Face	CLW *n* = 7 (10.4%)	Fractures *n* = 3 (4.5%)	Contusive trauma *n* = 1 (1.5%)	Teeth fracture *n* = 1 (1.5%)

Upper extremities	Clavicle fractures *n* = 8 (11.9%)		Contusive trauma *n* = 6 (8.9%)	

Chest	Rib fracture *n* = 4 (6.0%)		Contusive trauma *n* = 2 (3.0%)	Pneumothorax *n* = 1 (1.5%)

Abdomen	Contusive trauma *n* = 2 (3.0%)		Free intra-abdominal fluid *n* = 1 (1.5%)	

Lower extremities	Fractures *n* = 2 (3.0%)	Contusive trauma *n* = 3 (4.5%)		CLW *n* = 1 (1.5%)

External skin	Excoriation *n* = 3 (4.5%)			

*MBI: mild brain injury; **SAH: subarachnoid haemorrhage; ***CLW: contused lacerated wounds.
